# Ubiquitination of Neurotransmitter Receptors and Postsynaptic Scaffolding Proteins

**DOI:** 10.1155/2013/432057

**Published:** 2013-02-03

**Authors:** Amy W. Lin, Heng-Ye Man

**Affiliations:** Department of Biology, Boston University, 5 Cummington Mall, Boston, MA 02215, USA

## Abstract

The human brain is made up of an extensive network of neurons that communicate by forming specialized connections called synapses. The amount, location, and dynamic turnover of synaptic proteins, including neurotransmitter receptors and synaptic scaffolding molecules, are under complex regulation and play a crucial role in synaptic connectivity and plasticity, as well as in higher brain functions. An increasing number of studies have established ubiquitination and proteasome-mediated degradation as universal mechanisms in the control of synaptic protein homeostasis. In this paper, we focus on the role of the ubiquitin-proteasome system (UPS) in the turnover of major neurotransmitter receptors, including glutamatergic and nonglutamatergic receptors, as well as postsynaptic receptor-interacting proteins.

## 1. Introduction

Neurons are highly complex cells that form a network of connectivity throughout the brain via specialized structures called synapses. The human brain contains approximately 85–100 billion neurons that can generate an estimated 100 trillion synapses [1]. These synapses maintain a careful balance between network plasticity and stability through finely controlled mechanisms such as intracellular trafficking and posttranslational modification of synaptic proteins. One such modification is ubiquitination, which is known to play a role in synaptic physiology and synapse formation, as well as in synaptic protein trafficking, stability, internalization, and degradation [2]. Malfunction of the ubiquitin system is also involved in the development of brain disorders such as autism, Alzheimer's disease, Huntington's disease, amyotrophic lateral sclerosis (ALS), and Parkinson's disease [3].

**Figure 1:**
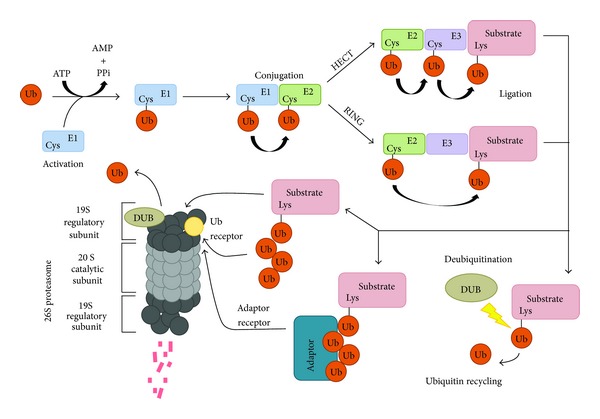
Enzymatic cascade leading to substrate ubiquitination. Three sets of enzymes are required for ubiquitination of a targeted substrate: ubiquitin-activating (E1), ubiquitin-conjugating (E2), and ubiquitin-ligase (E3) enzymes. There are two main classes of E3 enzymes, the RING and HECT classes, which differ in the manner by which they transfer Ub to a target substrate. Once a Ub molecule is conjugated to its target protein, additional Ub molecules can be attached to form chains (see Figure 2 for a more detailed illustration of Ub binding). However, since ubiquitination is a reversible process, once Ub is attached, deubiquitinating enzymes (DUBs) can then hydrolyze the isopeptide bond between Ub and its target protein (shown by the small lightning bolt) and thus return the protein to its previous state and release Ub. Substrates that contain polyUb chains are often targeted to the proteasome, where they are bound and subsequently degraded. The proteasome is composed of a catalytic 20S core particle structure and two 19S regulatory caps which together are collectively termed the 26S proteasome. While some polyubiquitinated proteins can be bound directly through polyUb binding subunits on the proteasome, others must be shuttled to the proteasome via adaptor proteins (the binding site for Ub and adaptors is represented by a yellow circle). Once the substrate is bound to the proteasome, many ATPase subunits that make up the proteasome utilize ATP to unfold the protein, simultaneously deubiquitinating the protein and releasing Ub while cleaving the protein into small peptide fragments.

 Ubiquitin (Ub) is a small, highly conserved protein expressed in all eukaryotic cells that modulates an extensive range of biological functions including DNA repair, transcription, endocytosis, autophagy, and protein degradation. Structurally, ubiquitin is an 8.5 kDa, 76 amino acid polypeptide that forms a compact structure with an exposed carboxy terminal tail containing a diglycine motif that can be covalently ligated via an isopeptide bond to the primary *ε*-amino group of lysine (Lys) residues on a target substrate. This occurs through an enzymatic process termed ubiquitination, a reversible posttranslational modification that can affect the structure and activity of the targeted protein, along with its localization and binding interaction to other partners. The first step of this pathway involves the biochemical priming of ubiquitin by the E1 ubiquitin-activating enzyme (E1) in an ATP-dependent manner. E1 initially binds both Mg^2+^ and ubiquitin to form an unstable intermediate ubiquitin-adenylate species before ubiquitin is subsequently transferred to the catalytically active cysteine of E1, which results in a thiol-ester bond between the E1 cysteine and the carboxy-terminal glycine (G76) of Ub [4, 5]. Once a ubiquitin molecule is activated by E1, it is then accepted by an E2 ubiquitin-conjugating enzyme (E2), also through a thiol-ester bond between the cysteine of E2 and G76 of Ub [6]. E2 then goes on to form a complex with an E3 ubiquitin ligase enzyme (E3), which accepts the ubiquitin molecule from E2 in order to catalyze its conjugation to a Lys residue on target substrates (Figure 1). Of the enzymes involved during ubiquitination, it is the E3 ligase that confers substrate specificity.

There are approximately 500 E3 ligases estimated from the human genome [7]. The E3 family of ligases is divided into two classes based on whether they contain the conserved homologous to E6-AP (HECT) or really interesting new gene (RING) domains. The two classes differ mainly in how ubiquitin is transferred to the substrate. HECT E3 enzymes contain a catalytic cysteine that accepts Ub from E2 enzymes before Ub is transferred to the Lys residue of the target protein. RING E3s on the other hand act as scaffolds that facilitate E2 and substrate interaction in order to transfer Ub from E2 to the target substrate [8]. Ubiquitination is a reversible modification mediated by deubiquitinating enzymes (DUBs) that hydrolyze Ub-protein isopeptide bonds. There are over 100 DUBs that have been identified, which are categorized into five distinct subclasses. The original two classes of DUBs include ubiquitin C-terminal hydrolases (UCHs), which are believed to interact with monoubiquitinated proteins, and ubiquitin-specific proteases (USPs) that appear to act primarily to disassemble polyubiquitin chains [9]. The newer classifications include ovarian tumor proteases (OTUs), Josephin proteases, and JAB1/MPN/Mov34 metalloenzymes (JAMMs) [10]. While the collective progression of ubiquitination and deubiquitination of a target protein results in a number of possible cellular processes, the ultimate result of synaptic protein ubiquitination is generally removal and eventual degradation of the targeted protein (Figure 1). Ubiquitinated proteins are usually directed to the proteasome or lysosome for degradation. For membrane proteins including neurotransmitter receptors, ubiquitination leads to protein internalization, after which the receptors will be sorted either to recycling endosomes for reinsertion, or to the proteasome or lysosome for degradation. 

**Figure 2:**
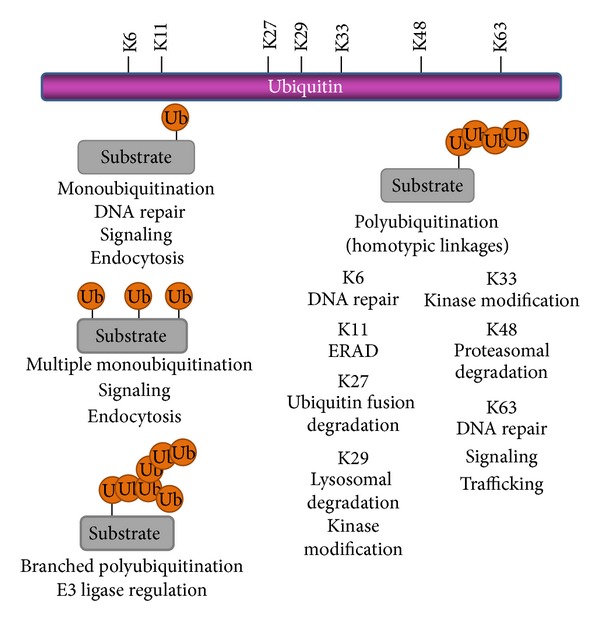
A schematic representation of the internal lysine residues available for Ub modification on ubiquitin as well as the different types of Ub modifications and their functional roles in the cell.

 The consequences of ubiquitination come in many flavors (i.e., monoubiquitination, multi-monoubiquitination, and polyubiquitination) depending on the number of ubiquitin moieties conjugated to the target and the specific type of Ub conjugation. The attachment of a single ubiquitin protein to one site (monoubiquitination, monoUb) or the attachment of single Ub molecules to multiple sites of a protein (multi-monoubiquitination) is generally involved in signaling, endocytosis, and subcellular localization. Additionally, ubiquitin itself can undergo ubiquitination to form distinct isopeptide-linked ubiquitin chains (polyubiquitination, polyUb) on any of its seven internal lysine residues (K6, K11, K27, K29, K33, K48, and K63), each of which can individually regulate a wide range of effects such as DNA repair, proteasomal or lysosomal degradation, and protein trafficking (Figure 2) [11]. To further complicate matters, atypical and forked Ub chains can also be formed [12]. Collectively, the activity of this one small protein is responsible for the regulation of multiple aspects of cellular signaling and function. 

 The synapse is the most fundamental and dynamic neuronal structure. In the brain, the majority of excitatory synaptic transmission is mediated by glutamate receptors, including AMPA receptors (AMPARs), NMDA receptors (NMDARs), and kainate receptors (KARs), whereas inhibitory transmission is mediated by GABA_A_ receptors (GABA_A_Rs). Through their intracellular domains, neurotransmitter receptors interact with multiple postsynaptic density (PSD) proteins. Glutamate and GABAergic receptors are highly mobile, trafficking constantly between the plasma membrane and cytosolic compartments. These dynamic processes, together with regulated receptor protein turnover, play a crucial role in synaptic plasticity and brain function. Although the PSD is a biochemically stable structure, its molecular architecture is highly responsive to changes in synaptic activity. Neuronal activity can cause ubiquitin proteasome system-(UPS-) dependent alterations in neurotransmitter receptors, as well as the composition and turnover of PSD proteins [13]. An increasing amount of research has shown that the UPS targets a wide range of neuronal proteins including both receptors and receptor-associated PSD proteins.

## 2. Ubiquitination of Postsynaptic Scaffolding Proteins

### 2.1. PSD-95

**Figure 3:**
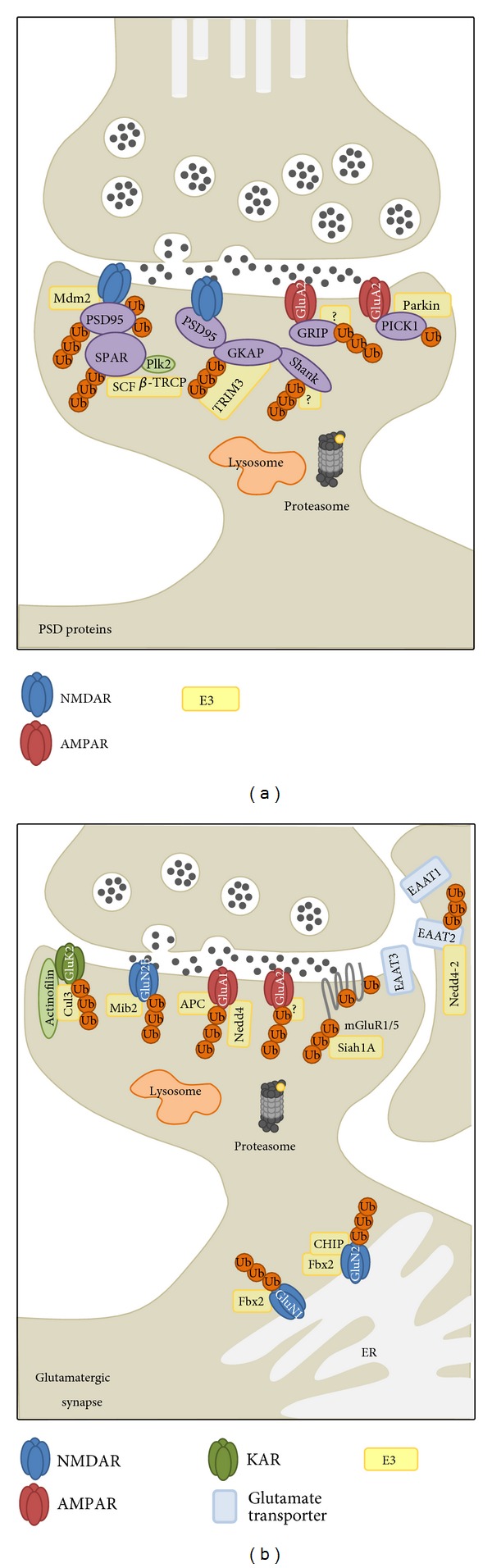
Postsynaptic ubiquitination. (a) Postsynaptic density proteins that undergo ubiquitination. PSD-95 is ubiquitinated by Mdm2. It is also monoubiquitinated at multiple sites and may be polyubiquitinated under specific circumstances. SPAR ubiquitination is reliant upon activity-dependent phosphorylation by Plk2 and regulated by the E3 complex SCF^*β*-TRCP^. Stimulation of neuronal activity can also cause TRIM3-mediated ubiquitination of GKAP. GKAP forms a scaffolding complex with Shank and GRIP, which are also ubiquitinated although their specific E3 ligases remain unidentified. PICK1 ubiquitination is mediated by parkin, which monoubiquitinates PICK1 to potentially regulate its downstream effects. (b) Ubiquitinated proteins at glutamatergic synapses in the mammalian system. mGluR1 and mGluR5 can be ubiquitinated by the E3 ligase Siah1A, which binds to a site in the C-terminus of both mGluRs. In mGluR5, Siah1A has been shown to mediate ubiquitination at multiple lysine residues. In the case of KARs, actinofilin acts as a scaffold to bind GluK2-containing KARs to the E3 ligase Cul3. During NMDAR assembly in the ER, glycosylated GluN1 is ubiquitinated by Fbx2, which also can recognize and ubiquitinate GluN2A. Fbx2 can also couple with other cochaperones such as CHIP to regulate GluN2A ubiquitination. GluN2B on the other hand, is localized to the PSD and ubiquitinated by Mib2. The GluA1 subunit of AMPARs has recently been shown to be ubiquitinated by Nedd4-1 primarily at K868. Another member of the Nedd4 family, Nedd4-2, has been shown to ubiquitinate the glial glutamate transporter EAAT2. Another recently identified E3 ligase for GluA1 is APC. As of yet, no E3 ligase has been identified for GluA2.

The postsynaptic density protein PSD-95 is an important scaffolding molecule regulating the localization of NMDARs and AMPARs [14] at the postsynaptic membrane. PSD-95 is known to be ubiquitinated by the E3 ligase Mdm2 (murine double minute) and degraded via the ubiquitin proteasome pathway in response to NMDA receptor activation (Figure 3(a)) [15]. Mutations blocking PSD-95 ubiquitination, as well as proteasomal inhibition, both effectively prevented ubiquitination-mediated PSD-95 degradation and were sufficient to block the internalization of AMPARs induced by direct stimulation of NMDARs [15]. Direct stimulation of AMPARs led to a decrease in PSD-95 expression, while overexpression of PSD-95 was correlated with a reduction in AMPAR endocytosis [16]. PSD-95 appears to be monoubiquitinated on multiple lysines [17] in response to a brief 10-minute treatment with NMDA [15]. Interestingly, PSD-95 ubiquitination results in an increased interaction with the *β*-adaptin subunit of the clathrin adaptor protein complex AP-2 that is Mdm2 dependent [17], suggesting the interesting possibility that PSD-95 ubiquitination may function as a signal for recruiting AP-2 to the postsynaptic membrane and subsequent AP-2-mediated AMPAR internalization [18]. Likewise intriguing is that a reduction in Cdk5 (cyclin-dependent kinase 5) activity has been shown to increase Mdm2-mediated PSD-95 ubiquitination without a subsequent decrease in PSD-95 levels, indicating a potential nonproteolytic signaling function for PSD-95 ubiquitination [17].

### 2.2. GRIP1

The glutamate receptor interacting protein 1 (GRIP1), a scaffolding protein that binds directly to GluA2 [19] and tethers AMPARs to other signaling proteins, is another target of ubiquitination (Figure 3(a)). Stimulation with glutamate downregulated levels of GRIP1 along with surface expression of GluA2, which can be blocked by inhibition of proteasome activity by MG-132 and the NMDAR antagonist MK-801, but not the AMPAR antagonist CNQX or EGTA, suggesting that glutamate-induced GRIP1 proteasomal degradation is mediated through an NMDAR and Ca^2+^ pathway [20]. As of yet, an E3 ligase for GRIP1 has not been identified.

### 2.3. GKAP and Shank

The Shank family of scaffolding proteins has three known members (Shank1, 2, 3) and binds to the GKAP (guanylate kinase-associated protein) scaffolding protein via Shank's PDZ domain. By binding to PSD-95 and other scaffolding proteins such as GRIP1 and Homer, GKAP and Shank fulfill a role as “master scaffold” proteins holding NMDARs, mGluRs, and AMPARs together as a super-complex [21]. Protein levels of GKAP and Shank in the PSD are activity regulated, leading to prominent ubiquitination of both proteins (Figure 3(a)) [13]. Stimulation of neuronal activity causes the E3 ligase TRIM3 (tripartite motif-containing protein 3) to stimulate ubiquitination and proteasome-dependent degradation of GKAP, causing a subsequent reduction of GKAP and Shank from the PSD [22]. RNAi against TRIM3 on the other hand prevents synaptic activity-induced loss of GKAP and results in an upregulation of GKAP and Shank, along with an enlargement of dendritic spines [22]. Interestingly, in a mouse genetic model of autism, expression of a single copy of a Shank3 C-terminal deletion mutation results in increased polyubiquitination of both Shank3 and NMDAR GluN1 and a subsequent reduction of GluN1 [23].

### 2.4. PICK1

Protein interacting with C-kinase 1 (PICK1) is a synaptic scaffolding protein that interacts with the AMPAR GluA2 subunit [24] and other synaptic proteins such as transporters and ion channels [25]. Parkin, a protein directly linked to Parkinson's disease (PD), functions as an E3 ligase to PICK1. Parkin binds to PICK1 through a PDZ-mediated interaction to enable PICK1 monoubiquitination (Figure 3(a)) [26]. Interestingly, while parkin does not cause PICK1 degradation, monoubiquitination of PICK1 by parkin may regulate the effects of PICK1 on the acid-sensing ion channel (ASIC), which may contribute to the symptoms observed in PD, such as affected signaling leading to excitotoxicity and dopaminergic neuron loss [26]. 

### 2.5. SPAR

Spine-associated RapGAP (SPAR) is a multidomain postsynaptic protein that forms a complex with PSD-95 and NMDARs by interacting with the guanylate kinase-like domain of PSD-95 to regulate actin dynamics and control dendritic shape [27]. SPAR undergoes activity-dependent phosphorylation-mediated degradation by a serum-inducible serine/threonine kinase (SNK) [28], also known as polo-like kinase 2 (Plk2). In the presence of Plk2, SPAR physically associates with and is degraded by Skp1/Cul1/F-box *β*-TRCP (SCF^*β*-TRCP^), a multisubunit E3 ligase (Figure 3(a)) [29]. Disruption of the SCF^*β*-TRCP^ complex can prevent Plk2-dependent degradation of SPAR [29]. 

## 3. Ubiquitination of Glutamate Receptors

Glutamatergic synapses mediate the vast majority of fast excitatory neurotransmission in the brain. Glutamate receptors are separated into two groups: the metabotropic mGluRs and the ionotropic glutamate receptors consisting of AMPARs, NMDARs, and KARs. Given the importance of receptor accumulation at synapses, ubiquitination-dependent receptor trafficking and abundance is considered an important regulatory mechanism in synaptic plasticity (Figure 3(b)). In addition to glutamate receptors, the main glutamate transporter GLT-1/EAAT2 is shown to be ubiquitinated via the ubiquitin ligase Nedd4-2, which mediates the PKC-dependent ubiquitination and downregulation of GLT-1 [30–33].

### 3.1. mGluRs

The mGluRs are G-protein-coupled receptors (GPCRs) that belong to three groups consisting of mGluR1-8 and are divided by physiological activity. The group one mGluRs (mGluR1 and mGluR5) localize primarily to the postsynaptic membrane while the remaining two groups localize to the presynaptic sites. The RING family E3 ubiquitin ligase, seven in absentia homolog (Siah1A), binds to a site in the C-terminus of both mGluR1 and mGluR5 that can be competitively inhibited by Ca^2+^/calmodulin (CaM) in a Ca^2+^-dependent manner [34]. Siah1A-mediated degradation of the group one mGluRs is abolished by proteasomal inhibition, as well as by mutations in the RING-finger domain of Siah1A [35]. Subsequently, site-directed mutagenesis of mGluR5 lysine residues demonstrates that Siah1A-mediated ubiquitination can occur at multiple lysine residues [35]. A more recent study describes a novel interaction between the mGluR-interacting protein Homer-3 and the S8 ATPase regulatory subunit of the 26S proteasome. Thus, Homer-3 may serve as an adaptor shuttling ubiquitinated mGluR1*α* to the proteasome for degradation [36].

### 3.2. Kainate Receptors

KARs consist of GluK1–5 subunits. The GluK1–3 subunits can form both homomers and heteromers; however, GluK4 and GluK5 can only form functional channels in combination with GluK1–3. GluK2 is targeted by the Cullin 3 (Cul3) E3 ubiquitin ligase complex for ubiquitination and degradation. The specificity is guided by the adaptor protein actinofilin, which interacts with the E3 ligase and the C-terminus of GluK2 [37, 38]. It is interesting to note that GluK2 is also subject to modification by the small ubiquitin-like modifier protein (SUMO) [39], leading to receptor internalization. During KAR-mediated LTD, KARs are heavily affected by PKC-mediated phosphorylation GluK2 at serine 868, which promotes GluK2 SUMOylation at lysine 886 and the subsequent internalization of GluK2-containing KARs [40–42]. SUMOylation-induced GluK2 internalization promotes its binding with mixed lineage kinase-3 (MLK3), leading to the activation of the MLK3-JNK3 pathway that may be responsible for ischemic neuronal cell death [43].

### 3.3. NMDA Receptors

NMDARs are heterotetramers normally assembled from GluN1 and GluN2 subunits that come from four gene products (GluN2A-D). During assembly of NMDARs, any GluN1 subunits bound to high-mannose glycans are ubiquitinated by the neuron-specific F-box protein Fbx2 and degraded through the ERAD pathway, with overexpression of Fbx2 leading to enhanced ubiquitination of glycosylated GluN1 [44]. GluN2 NMDAR subunits can also be ubiquitinated. While Fbx2 can recognize GluN1 and GluN2A in different contexts, it may couple with other cochaperones such as CHIP (C-terminus of Hsp70-interacting protein) to regulate ubiquitination of specific NMDAR subunits, in this case GluN2A [45]. NMDAR GluN2B subunits on the other hand are ubiquitinated by the RING family E3 ligase Mindbomb2 (Mib2), which is localized to the PSD and directly interacts with and ubiquitinates GluN2B to downregulate NMDAR activity [46]. Phosphorylation by the Src-family protein-tyrosine kinase Fyn enhances the protein-protein interaction between Mib2 and GluN2B, and subsequently, the ubiquitination of GluN2B by Mib2 [46]. 

### 3.4. AMPA Receptors

AMPA receptors (AMPARs) play a critical role in mediating the majority of fast excitatory synaptic transmission in the brain, where alterations in receptor expression, distribution, and trafficking have been shown to underlie synaptic plasticity and higher brain function. AMPARs are heterotetrameric receptors containing subunits GluA1–4. Evidence from several studies has emphasized the importance of the UPS in mediating AMPAR receptor trafficking and synaptic strength both directly and indirectly. The first system to show evidence of direct AMPAR ubiquitination was in *C. elegans*, where GLR-1 is shown to be ubiquitinated *in vivo* [47]. Mutations of GLR-1 lysine residues demonstrate an increase in GLR-1 synaptic quantity while overexpression of ubiquitin not only decreases GLR-1 expression at the synapse but also the density of synapses containing GLR-1 [47]. In *C. elegans*, multiple ubiquitin ligases have been implicated in UPS-dependent regulation of AMPAR synaptic abundance, including the anaphase-promoting complex (APC) [48], CUL3/KEL-8 [49], and RPM-1 [50]. AMPAR abundance can be affected by other pathways leading to degradation [51–53].

 In a mammalian system, it was observed that pre-treatment with proteasomal inhibitors completely and efficiently prevented glutamate-induced receptor internalization, indicating the requirement of UPS-dependent protein degradation in AMPAR trafficking [54]. However, although the UPS was shown to be recruited upon AMPAR activation in order to mediate AMPAR internalization, putative targets for degradation were believed to be proteins previously observed to interact with AMPARs, such as PSD-95 or other scaffolding proteins [13]. More recently, however, the mammalian AMPAR subunits GluA1 and GluA2 have been shown to be direct targets of ubiquitination [55–57]. Ubiquitination of GluA1 targets all four lysine residues at the intracellular GluA1 C-terminus, but mainly lysine 868 [57]. GluA1 ubiquitination is enhanced by glutamate but not NMDA application, indicating possible self-regulation of AMPAR amounts following AMPAR activation [55]. On the other hand, an increase of synaptic activity by application of the GABA_A_ antagonist bicuculline rapidly induces GluA2 ubiquitination, which is reversible by restoring basal neuronal activity [56]. Interestingly, in contrast to GluA1 where ubiquitination occurs mainly on the cell surface [57], GluA2 ubiquitination appears to occur after receptor endocytosis [56]. 

 Neural precursor cell expressed, developmentally downregulated (Nedd4) was identified as an E3 ligase in mammalian GluA1 ubiquitination [55, 57] along with APC, which can function as another E3 ligase [58]. However, neither Nedd4 nor APC is shown to be E3 ligases for GluA2 [55, 58]. A distinct E3 ligase for GluA2 likely exists in order to selectively ubiquitinate GluA2 subunits. AMPAR ubiquitination can be reversed by the protein deubiquitination enzyme USP-46 [59] and likely also AMSH (associated molecule with the SH3 domain of STAM) [60]. Thus far, ubiquitination of GluA3 or GluA4 has not been reported. 

## 4. Ubiquitination of Nonglutamate Receptors

### 4.1. GABA Receptors

GABARs mediate the majority of inhibitory neurotransmission in the brain. They are divided into two subclasses: ionotropic GABA_A_ receptors (GABA_A_Rs) and metabotropic GABA_B_ receptors (GABA_B_Rs). GABA_A_Rs are heteropentameric chloride channels assembled from a large selection of subunits (*α*1–*α*6, *β*1–*β*3, *γ*1–*γ*3, *δ*, *ε*1–*ε*3, *θ*, and *π*) which determine subsequent channel properties and localization [61].

**Figure 4:**
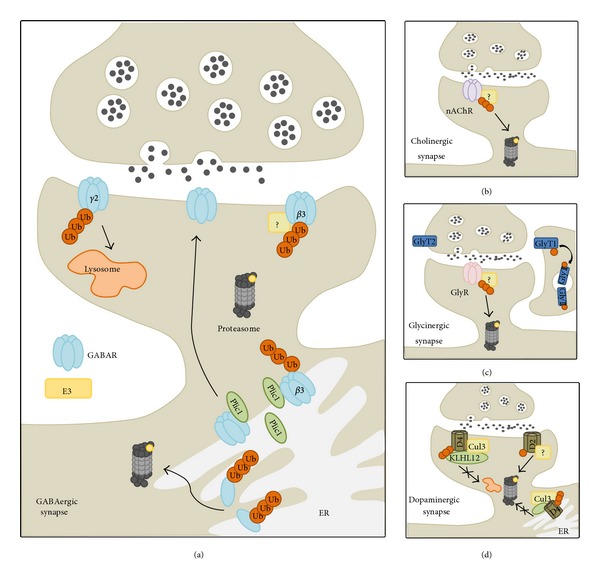
(a) GABAergic ubiquitination. The specific E3 ligase for GABARs has not been identified, although the ubiquitin-like protein Plic-1, which does not function for ubiquitination, directly interacts with GABA_A_Rs to affect insertion. It stabilizes polyubiquitinated GABA_A_Rs in the ER to limit ERAD and promote GABA_A_R surface expression. Unassembled GABAR subunits in the ER are usually ubiquitinated and targeted to the proteasome. Although the proteasome plays a large role in GABA_A_R trafficking, the lysosome also mediates GABA_A_R degradation by ubiquitinating a motif in intracellular domain of the *γ*2 subunit. (b) Cholinergic synapses. In the cholinergic synapse, the E3 ligases remain unidentified although it is known that ubiquitination of the *α*3, *β*2, and *β*4 nAChR subunits is required for degradation. (c) Glycinergic synapses. In glycinergic synapses, extensive ubiquitination to the *α*1 subunit of GlyR prior to internalization has been observed in Xenopus oocytes though this has not been repeated in a mammalian system. However, it has recently been shown that the glycine transporter GLYT1 1b subunit undergoes ubiquitination at lysine 619, causing rapid endocytosis. (d) Dopaminergic synapses. In dopaminergic synapses, KLHL12 acts as an adaptor to the E3 ligase Cul3 to promote polyubiquitination of both immature ER-associated and mature membrane-associated forms of the D4 receptor though there is apparently neither proteasomal nor lysosomal degradation observed. The D2 receptor subtype is known to be monoubiquitinated, although possible polyubiquitinated forms may also exist. An E3 ligase has yet to be identified.

 GABA_A_R ubiquitination (Figure 4(a)) is strongly regulated by neuronal activity. Chronic blockade of neuronal activity using tetrodotoxin (TTX) demonstrates a large increase in polyubiquitinated species of GABA_A_Rs and a resulting decrease in cell surface stability [62]. Conversely, an increase in neuronal activity enhances GABA_A_R stability by decreasing GABA_A_R ubiquitination [62]. Interestingly, ubiquitination appears to target mainly GABARs residing in the ER. Thus, increased polyubiquitination of GABA_A_Rs reduces receptor stability in the ER, leading to a reduction in receptor membrane insertion [62]. The mechanism underlying the proteasome-dependent loss of GABA_A_Rs following chronic activity blockade is still unclear but one possibility is through the L-type VGCCs (voltage-gated Ca^2+^  channels), which, when activated, alter the GABA_A_R turnover rate in a proteasome-dependent manner by regulating GABA_A_R insertion into the plasma membrane [63]. Studies find that the ubiquitin-like protein Plic-1, which does not function for ubiquitination, directly interacts with GABA_A_ receptors to facilitate GABA_A_ cell surface expression [64]. Plic-1 can stabilize polyubiquitinated GABA_A_Rs in the ER, reduce ERAD (endoplasmic reticulum-associated protein degradation), and promote GABA_A_R surface expression [65]. Although it appears that the proteasome is heavily involved in GABA_A_R trafficking, the lysosome has also been shown to mediate GABAR degradation. GABA_A_Rs are targeted to the lysosomal degradation pathway via the ubiquitination of a motif within the intracellular domain of the *γ*2 subunit [66]. For GABA_B_Rs, it has been shown that their degradation is enhanced by blockade of GABA_B_R recycling [67].

### 4.2. Acetylcholine Receptors

The nicotinic acetylcholine receptors (nAChRs) are heteropentamers, with the most common configuration as *α*4(2), *β*2(3) in the brain. Recent experiments in PC12 cells show that ubiquitination of the *α*3, *β*2, and *β*4 nAChR subunits are required for degradation (Figure 4(b)) [68]. Treatment with a proteasomal inhibitor demonstrates an increase in total subunit protein levels, as well as in fractions enriched for ER/Golgi, indicating a role for the ubiquitin pathway in nAChR trafficking. To date, the sites of ubiquitination and a potential E3 ligase remain unknown.

### 4.3. Glycine Receptors

Glycinergic receptors (GlyRs) are heteropentameric chloride channels consisting of multiple *α* (*α*1–*α*4) subunits and one *β* subunit [69]. In Xenopus oocytes, antagonist stimulation causes extensive ubiquitin conjugation to the *α*1 subunit of the GlyR prior to internalization, after which internalized GlyRs are proteolytically nicked into small fragments (Figure 4(c)) [70]. However, the function of GlyR ubiquitination remains unclear and it has not yet been shown in a mammalian system. Also, the E3 ligase(s) that targets GlyRs remains to be determined. In addition, it has recently been shown that the glycine transporter GLYT1 1b subunit undergoes ubiquitination at lysine 619, causing rapid endocytosis. This process can be stimulated by the PKC activator phorbol 12-myristate 13-acetate [71]. 

### 4.4. Dopamine Receptors

Dopamine receptors (DARs) are GPCRs subdivided into two groups: D1-type (D1 and D5) and D2-type (D2, D3, and D4). The D4 receptor has been associated with attention deficit hyperactivity disorder and possesses an interesting polymorphism in its third intracellular loop. KLHL12, a BTB-Kelch protein, can specifically bind to this region and act as an adaptor to a Cullin 3-based E3 ubiquitin ligase, thus promoting polyubiquitination of the D4 receptor (Figure 4(d)) [72, 73]. Ubiquitination assays of D1, D5, and D2L show that DAR subtypes other than D4 can undergo basal ubiquitination, although KLHL12 appears to function solely as an adaptor for D4 ubiquitination [72]. Further studies show that KLHL12 interacts with and promotes the ubiquitination of both immature ER-associated and mature plasma membrane-associated D4 receptors [73]. Surprisingly, experiments show that neither proteasomal ERAD degradation of new receptors nor lysosomal degradation of mature receptors occurs, an indication that GPCR ubiquitination may not always lead to degradation [73]. Another study found that the D2 receptor subtype can be monoubiquitinated at lysine 241 in the absence of an agonist (Figure 4(d)). It is interesting to note that the ubiquitination pattern of a generated K241A mutant differs distinctly from that of the wild-type D2 receptor, suggesting that the loss of the K241 site may promote ubiquitination of other lysine residues, thus rendering the protein more susceptible to degradation via the proteasome. This may be responsible for the observed reduction of mutant K241A membrane-associated DARs [74]. 

## 5. Conclusions and Future Perspectives

A stable amount of synaptic proteins results from a balance between protein synthesis and degradation. Whereas protein production can be controlled at multiple levels including gene regulation, transcription, mRNA stability, and translation initiation and efficiency, the regulatory mechanism for protein degradation is known mainly to be by ubiquitination. An ultimate consequence of synaptic protein ubiquitination that affects the abundance of PSD proteins and neurotransmitter receptors is alterations in synaptic strength. Thus, protein ubiquitination is believed to be a fundamental measure for the expression of synaptic plasticity via targeting on presynaptic transmission release, receptor abundance and spine stability and synapse formation [3, 75, 76]. Despite an increasing number of synaptic proteins found to be targets of ubiquitination, participating molecular elements and regulatory mechanisms are mostly unclear. In addition to the identification of contributing ubiquitination and deubiquitination enzymes, how neuronal activity is coupled to the translocation, activation, and deactivation of the enzymes and degradation machinery needs to be elucidated. Furthermore, dysfunction in protein degradation is a hallmark for neurodegenerative disorders such as Alzheimer's disease, Parkinson's disease, and ALS. Because synaptic function is often an important part of the pathology of these diseases, it is intriguing whether synaptic malfunction and protein aggregation result from the same defects in the UPS.
